# Health-related quality of life in individuals with genital herpes: a systematic review

**DOI:** 10.1186/s12955-022-01934-w

**Published:** 2022-02-16

**Authors:** Angela Devine, Xiuqin Xiong, Sami Lynne Gottlieb, Maeve Britto de Mello, Christopher K. Fairley, Jason J. Ong

**Affiliations:** 1 Global and Tropical Health Division, Menzies School of Health Research, Charles Darwin University, Darwin, Northern Territory Australia; 2 Centre for Epidemiology and Biostatistics, Melbourne School of Population and Global Health, University of Melbourne, Victoria, Australia; 3 Centre for Health Policy, Melbourne School of Population and Global Health, University of Melbourne, Victoria, Australia; 4 Global HIV, Hepatitis and STI Programmes, World Health Organization, Geneva, Switzerland; 5 Central Clinical School, Monash University, Melbourne, Victoria Australia; 6 Faculty of Tropical and Infectious Diseases, London School of Hygiene and Tropical Medicine, London, UK; 7 Melbourne Sexual Health Centre, Carlton, VIC 3053 Australia

**Keywords:** Herpes simplex virus, Quality of life, Systematic review, Genital herpes

## Abstract

**Background:**

There is a significant global burden of herpes simplex virus (HSV) related genital ulcer disease yet little is known about its impact on quality of life. This systematic review aimed to identify studies that quantitatively evaluated the effect of genital herpes on various aspects of health-related quality of life.

**Methods:**

Six databases were searched (MEDLINE, EMBASE, NHS Economic Evaluation Database, Health Technology Assessment, Database of Abstracts of Reviews of Effects, Web of Science Core Collection) for primary quality of life and economic evaluations of genital herpes from January 1, 2000 to January 7, 2021. Qualitative studies or those without primary data were excluded. Two authors independently extracted data from the publications. The study’s registration number with PROSPERO was CRD42021239410.

**Findings:**

We identified 26 relevant publications: 19 presented primary quality of life data, and seven were economic evaluations. The primary studies presented a range of condition-specific tools for describing the quality of life in individuals with genital herpes, but only one study used a direct valuation that could be used to generate utility weights. All economic evaluations of HSV infection were from high-income country settings. Most (6 of 7) focused on neonatal HSV infection with utilities adopted from studies prior to 2000.

**Interpretation:**

The extant literature on genital herpes-related quality of life is limited and requires updating. We recommend future studies be conducted in geographic- and population- diverse settings, and use preference-based condition-specific or generic-instruments to better inform economic modelling.

**Supplementary Information:**

The online version contains supplementary material available at 10.1186/s12955-022-01934-w.

## Introduction

An estimated 187 million people aged 15–49 years experienced at least one episode of herpes simplex virus (HSV) related genital ulcer disease in 2016 [1], and 491 million people aged 15–49 living with HSV-2 worldwide [2]. HSV-2 is a sexually transmitted infection that is lifelong, incurable, and can cause recurrent genital ulcer disease and neonatal herpes. Vertically transmitted neonatal herpes is associated with severe morbidity (e.g. long term neurodevelopmental disability) and mortality [3–5]. Genital HSV-2 infection can also increase HIV acquisition and have a significant psychosocial impact on those with the infection.

Given the large burden of HSV-related infection and disease and the lack of available interventions with population prevention impact, the development of vaccines against HSV is an important goal for global sexual and reproductive health [6]. No licensed HSV vaccines currently exist. Over the past decade, several HSV vaccine candidates have been evaluated in early clinical trials [7]; however, the development pipeline has slowed in recent years. Further information on the potential value of HSV vaccines in terms of their benefits for sexual and reproductive health will be helpful in decision-making related to advancing HSV vaccine development.

Information on the effect of genital herpes on quality of life is needed to determine health state values for decision analytic models; however, herpes-related quality of life has not been well-characterised in many settings globally, particularly in low- and middle-income countries (LMICs). These models are used in cost-effectiveness analyses to enable the efficient allocation of resources. Cost per quality-adjusted life years (QALY) is one popular outcome for cost-effective analyses. QALYs combine life-expectancy and its corresponding health-related quality of life, which reflects the impact of mortality and morbidity and can be used to compare across various conditions and interventions [8]. If quality of life information is not available, then cost-effectiveness analyses may need to look at other outcomes, such as HSV infections averted, which are not comparable across disease areas.

In addition to understanding the effects of herpes on quality of life, it is also important to review the instruments available to measure various aspects of quality of life in people living with herpes. These include instruments related to sexual health and well-being, which have been or could be used and validated in populations with genital herpes. In this sense, this review can guide future studies to fill data gaps towards the accurate measurement of genital herpes-related quality of life.

To our best knowledge, a systematic review of health-related quality of life in genital herpes has not been undertaken previously. Therefore, the purpose of this systematic review was to identify studies that quantitatively evaluate the effect of genital herpes on various aspects of health-related quality of life and to summarize survey instruments and measurement scales that have been or could be used for measuring quality of life and health utilities in people with herpes.

## Methods

### Search strategy and selection criteria

We conducted a systematic review following guidelines in the Cochrane Handbook to identify studies that quantitatively measure the quality of life for people living with asymptomatic or symptomatic genital herpes [9]. We also included studies that evaluated the impact of the vertical transmission of herpes on the quality of life related to neonatal herpes (both from the mother and neonate’s perspectives). The inclusion criteria were studies containing primary data associated with the quality of life in people with herpes, including randomized controlled trials, observational studies, economic evaluations, and primary valuation studies for health utilities. We excluded qualitative studies or those without primary data. Six databases were searched (MEDLINE, EMBASE, NHS Economic Evaluation Database, Health Technology Assessment, Database of Abstracts of Reviews of Effects, Web of Science Core Collection) on January 7, 2021. The search limits were from 2000-current, humans, and English language (full search strategy in Additional file 1: Appendix pp 2–10). Grey literature was also searched using OpenGrey for potentially useful information. Duplicated articles were excluded using Endnote X9.

We manually searched the reference lists of potentially relevant studies to identify additional studies, and further studies were also added based on our knowledge of the literature. Since few articles were found with our search, articles identified through the reference lists included papers published before 2000. This study was reported following the latest Preferred Reporting Items for Systematic review and Meta-Analysis Protocols (PRISMA) [10]. The study’s registration number with PROSPERO was CRD42021239410 (available from https://www.crd.york.ac.uk/prospero/display_record.php?RecordID=239410).

The studies were first screened by two reviewers independently (AD, XX), who reviewed all the papers' titles and abstracts and selected the studies that met the inclusion criteria. This process was conducted in Covidence. Any discrepancies were resolved by a third author (JO). Secondly, two reviewers (AD, XX) independently evaluated the full texts of all the selected articles. Abstracts whose full text could not be found but contained useful information were kept for data extraction. All the studies included for data extraction were classified into two groups: economic evaluation studies and primary valuation studies.

### Data analysis

For economic evaluations, data were collected about the study characteristics (author, publication year, year of data collection, country setting), the study population (age, number of participants), the health states and their utility or disutility values, the source of the health utilities, duration of health state, and type of sensitivity analysis used. For primary studies, data were collected about the study characteristics, the study population, methodology used to obtain the utilities (e.g. time-trade off, standard gamble, discrete choice experiments), the health states and their utility or disutility values, and the duration of the health state. The quality assessment of included primary studies was evaluated using a previously developed critical appraisal checklist for health-related quality-of-life studies by Picot and colleagues [11], while the quality assessment of economic evaluation studies was evaluated using the methods section of the CHEERS checklist[12] by one researcher. The initial assessment was split between two researchers (AD, XX) with a random 30% checked by a second reviewer (JO). Descriptive statistical analysis was conducted to summarise the study characteristics. Meta-analysis was not conducted because the quality-of-life data came from different instruments with different scales and scoring systems.

## Results

**Fig. 1:**
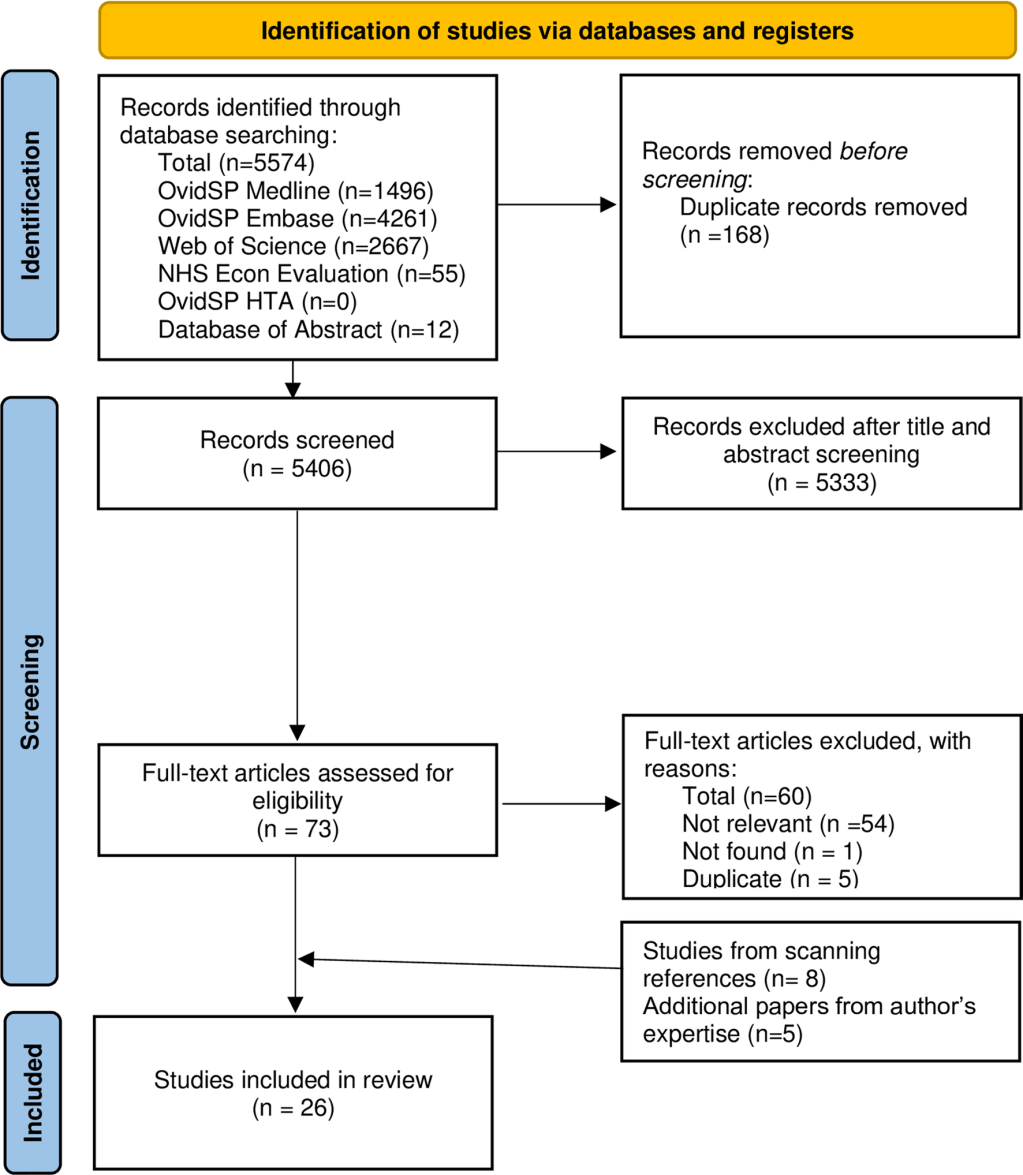
PRISMA flowchart

Our search identified a total of 5,406 unique studies; of these, 5,333 were excluded while 73 full texts were assessed for eligibility. After excluding 60 studies and adding 8 studies from scanning references, 21 studies related to genital herpes health-related quality of life were identified. The reasons for the exclusion of studies included not found (n = 1), not relevant (only abstract, no quality of life or utility data, review study) (n = 54), and duplication (n = 5). An additional five studies were added from the authors’ familiarity with the literature [13–17]. In total, we included seven economic evaluations and 19 primary studies evaluating the health-related quality of life of persons living with genital herpes. Tables 1 and 2 summarise the key demographics of the studies included. Figure 1 presents the PRISMA flowchart.

**Table 1:** Overview of included economic evaluation studies

Primary studies	Total (*N* = 19)
	n (%)
Country income level*	
High	14 (88)
Middle	2 (12)
Global	3 (3)
*Populations*	
Adults	18 (95)
People living with HIV	1 (5)
*Publication year*	
2010 or before	12 (63)
2011–2021	7 (37)

**Table 2:** Overview of included primary studies evaluating health-related quality of life of people living with herpes

Economic evaluation studies	Total (*N* = 7)
	n (%)
Country income level*	
High	7 (100)
*Populations*	
Pregnant women and neonates	6 (86)
Patients with genital herpes	1 (14)
*Publication year*	
2010 or before	5 (71)
2011–2021	2 (29)

*As per the New World Bank current 2021 fiscal year [16]

### Economic evaluations of genital herpes

Table 3 summarises the characteristics of the seven economic evaluation studies. Six studies targeted interventions for pregnant women and neonates [4, 18–23], and one study focused on people with recurrent genital herpes [22]. Two studies examined the cost-effectiveness of serologic testing for HSV infection in pregnant women [4, 19]. Other studies estimated the cost-effectiveness of testing and treating HS.V infection in neonates with fever [20], of offering prophylactic acyclovir treatment to pregnant women [21], of including treatment of stigma of genital herpes treatment [22], of preventing vertical HSV transmission [18], and of routine antenatal screening for HSV infection [23]. All the economic evaluation studies include QALYs as results. All studies conducted sensitivity analyses.

**Table 3:** Description of economic evaluations included in the review

Lead author	Evaluation aims	Year	Country	QALYs	Perspective	Population	Health States included for HSUVs	Sensitivity analysis	Source for HSUVs
Baker [17]	Determine whether serologic testing for herpes simplex virus type 2 (HSV-2) in pregnant women and their partners is cost-effective	2004	USA	Yes	Societal	Pregnant women	Neonatal herpes infection (mild, moderate, severe, normal or no permanent impairment)	One-way and multiway	[22]
Caviness [18]	Determine the clinical effectiveness and cost-effectiveness of testing for and empirically treating HSV infection in neonates with fever aged from birth to 28 days	2008	USA	Yes	Societal	Febrile neonates	Neonatal herpes infection (normal, mild, moderate, severe, death)	One-way, probabilistic sensitivity analysis using Monte Carlo simulation	[22]
Chatroux [4]	Estimate whether serotyping women with a history of genital HSV and an outbreak during the third trimester of pregnancy is cost-effective compared with no serotyping	2021	USA	Yes	Societal	Pregnant women	Neonatal HSV infection (mild, moderate, severe, death) From neonatal and maternal perspective	One-way, probabilistic sensitivity analysis using a Monte Carlo simulation	[22]
Little [19]	Addresses whether it would be clinically beneficial and cost-effective to offer prophylactic acyclovir to women with a history of HSV but no recurrence during pregnancy	2005	USA	Yes	Health care	Pregnant women	Neonatal HSV infection (neonatal perspective: moderate disability, severe disability) (maternal perspective: caesarean delivery, having an impaired child, losing a child)	One-way, probabilistic sensitivity analysis using a Monte Carlo simulation	[23–25]
Smith [20]	Investigate the effect of including treatment of disease stigma on the cost-effectiveness of genital herpes treatment	2000	USA	Yes	Societal	Adults with genital herpes	Genital herpes	Multiple sensitivity analyses	Not stated
Tuite [16]	Assess the effectiveness and cost-effectiveness of identifying pregnant women at risk of de novo HSV acquisition as a means of preventing vertical HSV transmission	2011	Canada	Yes	Unclear	Pregnant women	Neonatal HSV infection (normal/mild, moderate, severe, death)	One-way, multi-way, probabilistic sensitivity analysis	[26]
Thung [21]	Determine the cost-effectiveness of routine antenatal screening for herpes simplex virus 1 and 2 in women without a known history of genital herpes	2005	USA	Yes	Health care	Pregnant women	Neonatal HSV infection (normal, mild, moderate, severe)	One-way	[27]

*HSUV* health-state utility values; *HSV* Herpes simplex virus; *QALY* quality-adjusted life year

Table 4 summarises the utilities applied in the health states in the cost-effectiveness studies. All six studies evaluating neonatal HSV infections' health-related quality of life used health state utilities based on the final outcomes of neonatal HSV infection, e.g., mild, moderate or severe disability, and death. Two studies also applied the health state utilities of neonatal HSV infection from the maternal perspective, such as having an impaired child or losing a child [4, 21]. We only found one study focused on genital herpes that applied a disutility related to stigma and symptomatic recurrence of genital herpes [22].

**Table 4:** Summary of utility values included in economic evaluations for health states

Lead author	Neonatal HSV infection						Genital herpes		
	Neonatal perspective			Maternal perspective					
	HSUV	Duration (years)	Probability of health state	HSUV	Duration (years)	Probability of health state	HSUV	Duration (years)	Probability of health state
Baker [17]	No permanent impairment: 1	76.4	0.56						
	Mild: 0.82	76.4	0.05	··	··	··	··	··	··
	Moderate: 0.52	76.4	0.08	··	··	··	··	··	··
	Severe: 0.16	20	0.14	··	··	··	··	··	··
	Death: 0	.	0.17	··	··	··	··	··	··
Caviness [18]	Normal: 1	77.8	··	··	··	··	··	··	··
	Mild: 0.82	77.8	Differs according to treatment arm, time and disease state (e.g., 12-month outcome with acyclovir therapy for disseminated disease: Normal: 0.28, Mild: 0.04, Moderate: 0.02, Severe: 0.13, Death: 0.53)	··	··	··	··	··	··
	Moderate: 0.52	77.8		··	··	··	··	··	··
	Severe: 0.16	20		··	··	··	··	··	··
	Death: 0	.		··	··	··	··	··	··
Chatroux [4]	Mild: 0.82	79.3	HSV-1: 0.69; HSV-2: 0.49	Mild: 0.94	54.8	HSV-1: 0.69; HSV-2: 0.49	··	··	··
	Moderate: 0.52	79.3	HSV-1: 0.01; HSV-2: 0.14	Moderate: 0.87	54.8	HSV-1: 0.01; HSV-2: 0.14	··	··	··
	Severe: 0.16	20	HSV-1: 0.02; HSV-2: 0.17	Severe: 0.76	54.8	HSV-1: 0.02; HSV-2: 0.17	··	··	··
	Death: 0	··	HSV-1: 0.28; HSV-2: 0.2	Death: 0.92	54.8	HSV-1: 0.28; HSV-2: 0.2	··	··	··
Little [19]	Normal neonate: 1	77.2	#	Caesarean delivery: 0.99	55.4	If lesions present at delivery: 1; if no lesions present: 0.244	··	··	··
	Moderate: 0.9	62.0	HSV-1: 0.01, HSV-2: 0.14	Having an impaired child: 0.81	55.4	Not reported	··	··	··
	Severe: 0.3	28.7	HSV-1: 0.01, HSV-2: 0.17	Losing a child: 0.92	55.4	Not reported	··	··	··
	Death: 0	··	HSV-1: 0.28, HSV-2: 0.20	··	··	··	··	··	··
Smith [20]	··	··	··	··	··	··	Stigma: 0.95	10	Unclear
	··	··	··	··	··	··	Symptomatic recurrence: 0.90	10	
Tuite [16]	Normal/mild: 1	75	Unclear	··	··	··	··	··	··
	Moderate: 0.84	38	Unclear	··	··	··	··	··	··
	Severe: 0.41	38	Unclear	··	··	··	··	··	··
	Death: 0	··	Unclear	··	··	··	··	··	··
Thung [21]	Normal: 1	76	Unclear	··	··	··	··	··	··
	Mild: 1	76	Unclear	··	··	··	··	··	··
	Moderate: 0.5	76	Unclear	··	··	··	··	··	··
	Severe: 0.1	76	Unclear	··	··	··	··	··	··
	Death: 0	.	Unclear	··	··	··	··	··	··

*HSUV* health-state utility values; *HSV* Herpes simplex virus; *QALY* quality-adjusted life years

The seven economic evaluations referenced other literature to obtain their utility weights, with four different sources for neonatal outcomes. Two sources directly valued neonatal HSV infection outcomes [24, 25], one source valued the health states of survivors born with extremely low birth weight [26], and one source did not have clear data on HSV related health states [27]. For the same health states, different sources reported different values. The utility for mild neonatal impairment ranged from 0.82 to 1, moderate impairment ranged from 0.5 to 0.9, and severe impairment ranges from 0.1 to 0.41. Notably, all the sources for the neonatal HSV infection outcomes came from studies published before 2000. All utility weights were applied for the duration of life expectancy. Two studies did not report any incidence or probability of developing different outcomes (mild, moderate, severe impairment).

### Primary studies

Nineteen primary studies with utility values for herpes were identified by the systematic review (Table 5). Four studies were conducted alongside clinical trials comparing episodic and suppressive therapy [28–31]. The rest of the studies were undertaken to develop herpes-specific quality of life measures [32], evaluate instruments to measure the quality of life in those with genital herpes [33, 34], examine sexual well-being or quality of life [13, 14, 34–38], and to measure psychosocial responses to a new HSV-2 diagnosis [39, 40].

**Table 5:** Summary of primary studies for measuring the quality of life of people living with herpes

Lead author	Study aims	Participants (n)	Country	Year(s) collected data	Techniques used, health states valued and results	Type of valuation
**Utility studies**						
Bartlett [26]	Assess time to first recurrence and change in RGHQoL for episodic and suppressive famciclovir	Immunocompetent adults with recurrent genital herpes (384)	USA	Published 2008*	**RGHQoL = mean** Episodic famciclovir at baseline = 30.7 Episodic famciclovir at the end of study = 33.5 Suppressive famciclovir at baseline = 29.3 Suppressive famciclovir at the end of study (30 days after the last dose) = 34.0	Indirect
Bodsworth [27]	Demonstrate that a 2-day course of famciclovir 500 mg stat, then 250 mg twice daily was non-inferior to the standard 5-day course of 125 mg twice daily	Immunocompetent adults with recurrent genital herpes (873)	Australia & Canada	2003–2006	**HSC = mean (SD)** 2-day famciclovir = 4.77 (3.76) 5-day famciclovir = 4.98 (3.83) **HOIQ = mean (SD)** 2-day famciclovir = 5.70 (5.12) 5-day famciclovir = 5.78 (5.29)	Indirect
Fife [28]	Compare episodic and suppressive therapy for genital herpes about disease characteristics, disease burden, and psychological impact	Healthy adults with a history of 4–9 genital herpes recurrences per year for at least one year (80)	USA	1999–2000	**SF-36** Not reported. (No difference between study arms.) **RGHQoL ~ mean (estimated from a figure)** Genital herpes with episodic treatment at baseline ~ 26 Genital herpes with episodic treatment at month 12 ~ 21 Genital herpes with suppressive treatment ~ 22.5 Genital herpes with suppressive treatment at month 12 ~ 15	Indirect
Fisman [31]	Evaluate the use of several utility-based tools for assessment of health-related quality of life in a population of individuals with symptomatic genital herpes	Individuals with recurrent genital herpes (39)	Canada	Published 2005*	**TTO: mean (SD)** Asymptomatic genital herpes = 0.89 (0.21) Symptomatic genital herpes = 0.89 (0.22) **VAS: mean (SD)** Asymptomatic genital herpes = 0.76 (0.30) Symptomatic genital herpes = 0.71 (0.30) **HUI-2: mean (SD)** Transient symptomatic = 0.93 (0.08) Asymptomatic = 0.80 (0.16) **RGHQoL: median (range)** Study population = 20 (1–57)	Direct
Foster [36]	Investigate the extent to which stigma experiences, individual characteristics, and STI characteristics were associated with multiple aspects of sexual well-being among individuals diagnosed with herpes and/or HPV	Individuals with herpes and/or human papillomavirus (188: 83 had herpes and 79 were co-infected)	Canada	Published 2016*	**Sexual Anxiety Inventory = mean (SD)** Individual with STI = 33.7 (11.1) **Sexual Self-Schema Scale-Revised** ‘moderately positive self-schemas’ = 129.5 (30.0) **Sexual Self-Esteem Scale** ‘moderately high levels of sexual self-esteem’ = 30.4 (7.3) **GMSEX-R (sexual satisfaction) = mean (SD)** Individual with STI = 25.2 (6.2)	Indirect
Langley [35]	Assess the impact of genital herpes and extragenital warts on current health-related quality of life at the general population level	Herpes patients obtained from a general population sample (520 with genital herpes, 63 were co-infected)	UK, France, Spain, Italy and Germany	2008	**SF-6D: mean + 95% CI** Genital herpes = − 3.52 (− 4.63 to – 2.71) External genital warts and genital herpes = − 5.00 (− 1.76 to – 8.25)	Indirect
Mehta [11]	Assess SQoL among heterosexual couples in Kisumu, Kenya, and how this was associated with HIV status, STIs, and sexual practices	252 couples (53% women were HSV-2 + , 47% men were HSV-2 +)	Kenya	2014–2017	**SQoL**^**2**^** = mean** Men = 87.7 (21.9) Women = 78.2(20.6) Note: mixed infection with HIV, HSV-2 and bacterial vaginosis	Indirect
Meyer [38]	Measure the psychosocial response to a new serological HSV-2 diagnosis in patients receiving care at an urban HIV clinic prospectively	HIV-infected adults, aged 18–55 (248)	USA	2001–2002	**25 herpes specific questions**^**1**^** = mean (SD)** HSV-2 + with previous history at 2 weeks = 73.7 (12.4) HSV-2 + with previous history at 3 months = 73.8 (12.6); HSV-2 + with previous history at 6 months = 75.4 (10.8); HSV-2 + without previous history at 2 weeks = 76.4 (14.0) HSV-2 + without previous history at 3 months = 75.0 (15.7) HSV-2 + without previous history at 6 months = 76.8 (14.4)	Indirect
Patel [39]	To investigate whether suppressive antiviral therapy improves health-related quality of life in patients with recurrent genital herpes	Participants in a randomised clinical trial comparing herpes therapy options (1349)	USA, UK, Denmark, France, Australia, New Zealand, Italy, Austria, & Germany	Published 1999*	**RGHQoL = mean score** Baseline = ranged from 30.2 to 33.9 depending on six treatment groups At 12 months, the adjusted mean improvement from baseline in the active treatment groups ranged from 9.4 to 12.0 compared with a mean improvement of 4.8 points in the placebo group	Indirect
Raj [12]	Compare the HRQOL in patients with HIV, genital HPV, and HSV-2 infections and healthy controls	HSV-2 + adults attending a sexually transmitted diseases clinic (60)	India	2008–2009	**WHOQOL-BREF = Mean (CI)** Physical domain (domain I) = 28.5 (24.8–32.2) Psychological domain (domain II) = 34.3 (30.5–38.1) Social domain (domain III) = 36.9 (31.5–42.3) Environmental domain (domain IV) = 41.3 (37.8–44.8) Total QOL score = 141.0 (128.1–153.9)	Indirect
Richards [37]	Measure the uptake of HSV-2 testing and psychosocial response to a new serologic diagnosis of HSV-2 in a health maintenance organisation (HMO) population prospectively	HSV-2 + adults, aged 18 + (87: 44 did not have a prior diagnosis)	USA	2002–2003	**HRQoL = mean score (SD)** HSV-2 + with prior diagnosis at 2 weeks = 77.2 (7.4) HSV-2 + with prior diagnosis at 3 months = 77.6 (8.5) HSV-2 + with prior diagnosis at 6 months = 77.4 (7.8) HSV-2 positive without prior diagnosis at 2 weeks = 74.9 (12.9) HSV-2 positive without prior diagnosis at 3 months = 79.8 (10.9) HSV-2 positive without prior diagnosis at 6 months = 77.6 (14.1)	Indirect
Romanowski [29]	Assess patients’ preference, satisfaction, and quality of life with suppressive versus episodic treatment of recurrent genital herpes	Immunocompetent adults (aged 18 +) with a documented history of genital HSV-1 or HSV-2 infection (225)	Canada	Published 2003*	**RGHQoL: Mean Score (95% CI)** Suppressive therapy week 12 = 59.6 (56.90 to 62.30) Suppressive therapy week 24 = 60.9 (58.32 to 63.54) Suppressive therapy week 36 = 59.9 (57.05 to 62.78) Suppressive therapy week 48 = 61.1 (58.29 to 63.86) Episodic therapy week 12 = 56.0 (53.22 to 58.72) Episodic therapy week 24 = 56.6 (53.71 to 59.45) Episodic therapy week 36 = 60.7 (57.56 to 63.77) Episodic therapy week 48 = 61.7 (58.70 to 64.69)	Indirect
Spencer [32]	Describes the impact of the disease on quality of life in a French population and used to provide additional validation data for the French version of the RGHQoL measure	Herpes patients obtained from a general population sample (150)	France	Published 1999*	**RGHQoL = mean (SD)** Men = 51.0 (6.5) Women = 50.8 (8.7)	Indirect
Taboulet [34]	Assess psychological morbidity in France related to genital herpes infection	Adults with recurrent genital herpes (150)	France	1995	**SF-36 = mean (SD)** physical functioning = 85.78 (20.82) physical role = 83.95 (29.88) bodily pain = 73.17 (25.41) general health = 68.39 (21.74) vitality = 55.37 (18.40) social functioning = 78.63 (21.54) emotional role = 76.01 (32.60) mental health = 60.75 (18.81) reported health = 47.47 (17.68)	Indirect
Wild [30]	Develop a herpes-specific quality of life measure	Adults with recurrent genital herpes (69)	USA	Published 1995*	**“25 Herpes-specific questions” = mean (SD)** mild severity of outbreak = 77.2 (16.1) moderate severity of outbreak = 67.4 (19.1) outbreaks in past 12 months = 82.1 (17.1) 2 + outbreaks in past 12 months = 72.1 (17.4)	Indirect
Wylomanski [33]	Test the hypothesis that vulvar disease patients have an overall impaired sexual well-being that varies depending on the type of vulvar disease	Patients with vulvar disease (72: 2 with vulvar herpes)	France	2011–2013	**FSFI = median (IQR)** Patients with VD (2 of 72 had vulvar herpes) = 21.1 (IQR: 13.4–26.5)	Indirect
James [13]^‡^	Quantify non-fatal health outcomes in terms of incidence, prevalence, and years of life with disability for a list of 354 Global Burden of Disease causes for the years 1990–2017	Unclear	Global	1990–2017	**Disability weight = base value (95% uncertainty interval)** Moderate infection due to initial genital herpes episode (infectious disease, acute episode, moderate) = 0.051 (0.032–0.074) Symptomatic genital herpes (infectious disease, acute episode, mild) = 0.006 (0.002–0.012)	Unclear
Salomon [14]^‡^	Estimate an updated set of disability weights for Global Burden of Disease 2013 via new data collection in four European countries and a reanalysis of existing and new data combined	Adults, aged 18 +	Global	2009–2010 and 2013	**Disability weight = base value (95% uncertainty interval)** Infectious disease: acute episode, mild = 0.006 (0.002–0.012) Infectious disease: acute episode, moderate = was 0.051 (0.032–0.074) Infectious disease: acute episode, severe = 0.133(0.088–0.190)	Unclear
Salomon [15]^‡^	Re-estimate disability weights for the Global Burden of Disease Study 2010 through a large-scale empirical investigation in which judgments about health losses associated with many causes of disease and injury were elicited from the general public in diverse communities through a new, standardised approach	Adults, aged 18 +	Global	2009–2011	**Disability weight = base value (95% uncertainty interval)** Infectious disease: acute episode, mild = 0.005 (0.002–0.011) Infectious disease: acute episode, moderate = 0.053 (0.033–0.081) Infectious disease: acute episode, severe = 0.210 (0.139–0.298)	Unclear

*sFSFI* Female Sexual Function Index; *GMSEX-R* Global Measure of Sexual Satisfaction; *HOIQ* Herpes Outbreak Impact Questionnaire; *HRQoL* Health-related Quality of Life; *HSC* Herpes Symptom Checklist; *HUI-2* Health utilities index; *MOS SF-36* Medical Outcome Study Short Form 36-item Health Survey; *QoL* Quality of Life; *RGHQoL* Recurrent Genital Herpes Quality of Life; *SQoL* Sexual Quality of Life; *STI* sexually transmitted infection; *TTO* time trade-off; *VAS* visual analogue scale; *WHOQOL-BREF* WHO Quality of Life instrument

^*^These studies do not state when data were collected

^‡^These are disutility studies

^1^These were scored on a 4-point scale

^2^SQoL was assessed with an 18-item female and 11-item male survey

There were 11 condition-specific instruments found to measure the genital herpes related quality of life, and Recurrent Genital Herpes Quality of Life (RGHQoL) was the most frequently used (6 studies used RGHQoL among total 19). The psychometric properties for these instruments were limited and variable in nature. For example, RGHQoL were shown to have good reliability [41], while Global measure of sexual satisfaction-revised (GMSEX-R) showed high internal consistency [38, 42]. There were some instruments (e.g. Sexual Self-Esteem scale) without any information of psychometric evidence. A more detailed summary of each condition-specific instrument used to measure the quality of life in people living with genital herpes is found in the Additional file 1: Appendix (pp 11–13).

The disability weights of HSV infection related health states came from the Global Burden of Disease (GBD) studies. The most recent GBD study (2017) had disability weights for ‘moderate infection due to initial genital herpes episode’ as 0.051 (0.032–0.074), and ‘symptomatic genital herpes’ as 0.006 (0.002–0.012) [15]. Previous GBD studies did not have disability weights specific to herpes. In GBD 2013, the disability weights for “infectious disease: acute episode, mild” was 0.006 (0.002–0.012) while moderate was 0.051 (0.032–0.074), and severe was 0.133 (0.088–0.308) [16]. The 2010 GBD had mild, moderate, and severe acute episodes as 0.005 (0.002–0.011), 0.053 (0.033–0.081), and 0210 (0.139–0.298), respectively [17]. The methods used to derive disutility weights have evolved over time, using data from surveys that began in 2009 using participants who were 18 years and older from a range of countries [16]. The most recent analysis presents 234 health states [15].

Most of the studies were focused on adult populations in Europe and North America except for the GBD studies and two other studies, one from India[14] and one from Kenya [13]. Of the instruments used to assess quality of life, the Recurrent Genital Herpes Quality of Life (RGHQoL) instrument was used the most frequently (six studies) [28, 30, 31, 33, 34, 43]. Many instruments were designed specifically for assessing quality of life in herpes infections and/or sexual health. The most recent study finished collecting data in 2017 [13], but many studies were much older. Six studies had less than 100 participants [14, 30, 32, 33, 35, 39].

The quality assessment of included studies is also found in the Additional file 1: Appendix (pp 14–23).

## Discussion

This systematic review synthesises the available evidence from published studies evaluating the effect of genital herpes on various aspects of health-related quality of life globally. It presents an overview of existing survey instruments and measurement scales that could measure the quality of life and health utilities in people with genital herpes. Only one small study from Canada used direct methods (time trade-off, visual analogue scale) to report a utility weight [33]. Using direct methods or multi-attribute utility instruments are important because they can be directly converted to a utility weight to generate QALYs used in economic modelling.

There is currently no consensus on what utility weights to use for genital herpes related economic evaluations. The source literature used for utility weights in the economic evaluations in this review was all for neonatal HSV infection and published before 2000. Given the paucity of primary studies using instruments that can generate utility weights, we recommend future studies to incorporate generic multi-attribute utility instruments (e.g., SF-6D, EQ-5D) alongside condition-specific instruments or scales (such as RGHQoL) to explore the correlation between these instruments further. This would provide valuable data on the sensitivity and responsiveness of generic utility measurement compared to condition-specific instruments [44, 45]. Furthermore, a greater diversity of people living with genital herpes is urgently needed for ensuring the health-related quality of life measurements are relevant for different populations. Most primary studies identified in our review were from Europe and North America. The way that populations and subpopulations value various health states can differ significantly across cultures[46] and might change over time.

Almost all primary studies identified by our review used condition-specific quality of life instruments. One example is the RGHQoL, which was the most frequent instrument. The RGHQoL is an instrument with 20 statements to assess the long-term impact on the individuals’ quality of life [47]. Patients respond to each statement by indicating the level of their own limitation on a 4-point Likert scale with scores ranging from 0 (maximum limitation) to 3 (minimum limitation). While condition-specific instruments like the RGHQoL are designed to capture the unique concerns related to genital herpes, the use of these instruments presents challenges if wanting to compare to other diseases in economic evaluations as they are not using a standard preference-based measurement. Only one primary study used direct methods to elicit preferences for a set of utility weights: a study of 39 individuals with recurrent genital herpes in Canada and is now dated (published in 2005) [33]. Accordingly, it is difficult to recommend any utility weights to inform economic evaluations. The best option may be to use the disutility weights from the most recent GBD study, but the methodology on these studies is opaque with sparse information on the survey design and study population.

The information available from the economic evaluations was also limited. First, most were carried out before 2010 and used utilities published before and around 2000, which is outdated. Second, the evaluations primarily focused on interventions targeting pregnant women; with only one economic evaluation for genital herpes [22]. Only two studies included the impact on the mother of having an impaired child or losing a child due to neonatal [4, 21]. This underscores the importance for future economic evaluations to include the impact of neonatal herpes on parents’ quality of life. Furthermore, other important issues such as stigma were only explicitly modelled in one study [22]. Stigma due to herpes infections is a significant source of psychological distress[48] and can also contribute to its spread by deterring disclosure to sexual partners.

Given the current limitations in genital-herpes-related quality of life data, several options exist to improve this knowledge base. Where data using a preference-based measure has not been collected, or the preference-based instrument is not available, a solution may be to “map” or “crosswalk” from other measures of health outcomes. However, directly obtaining utility weights is preferred compared to mapping [49]. Mapping methods are limited by their lack of overlap between the descriptive systems of two measures and do not solve the problem of inadequacy in the descriptive system of the generic measure [50]. Alternatively, developing condition-specific preference-based measures for HSV infection-related diseases have challenges of cross program comparability between different diseases [50], and should be seen only as a supplement to generic preference-based measures [50]. Multiple aspects of quality of life are impacted in people with genital herpes, including physical [51], psychological and social dimensions [52], such as altered perceptions of self-esteem, isolation, fear of rejection and/or gender-based violence, concerns about transmitting the disease, and depression [32]. This information could be used for choosing generic preference-based measurements and the development of condition-specific preference-based measurements.

The strength of this study is that it is the first systematic review to critically appraise the literature on quantitative measurements of the quality of life for individuals with genital herpes. It provides an overview of current knowledge that highlights many existing gaps and thus provides guidance for future research. Several limitations should be noted. Due to the sparse results, we could not conduct a meta-analysis to provide a pooled estimate of utility weight. In addition, the diversity of condition-specific instruments underscored that no consensus has been reached on how to measure the specific impact of genital herpes on quality of life. Lastly, our study excluded non-English literature, so we may have missed data from other geographically diverse populations.

In conclusion, this systematic review identified major gaps in how health-related quality of life for people living with genital herpes is measured. Specifically, there is an urgent need to determine the health-related quality of life for people living with genital herpes and its sequelae from more contemporary populations living in various countries. We also need a better understanding of the determinants and modifying factors of health-related quality of life for people living with genital herpes to provide essential information to support investment in HSV vaccine development.

## Supplementary Information

**Additional file 1:** Appendices.

## Data Availability

All relevant data is provided in this manuscript. Further details can be obtained by contacting the corresponding author.
