# Supplementary material for: Health-related quality of life in individuals with genital herpes: a systematic review
Source: Health Qual Life Outcomes. 2022 Feb 16;20:25. doi: 10.1186/s12955-022-01934-w (PMC8848826; doi:10.1186/s12955-022-01934-w)
Supplement: Supplementary file 1 — Additional file 1: Appendices. [file 12955_2022_1934_MOESM1_ESM.pdf]

APPENDIX

Health-related quality of life in individuals with genital herpes: a systematic review

Angela Devine, Xiuqin Xiong, Sami Lynne Gottlieb, Maeve Britto de Mellol, Christopher K. Fairley, Jason J. Ong

Correspondence to: Jason Ong, 580 Swanston Street, Carlton, Victoria, 3053, Australia

Phone: +613 9341 6200

Email: Jason.ong@monash.edu or Jason.Ong@lshtm.ac.uk

Contents

Search Methodology. 2

Survey instruments for evaluating genital herpes quality of life 11

Female Sexual Function Index (FSFI) 11

Herpes Outbreak Impact Questionnaire (HOIQ) 11

Herpes Symptom Checklist (HSC) 12

Recurrent Genital Herpes Quality of Life (RGHQoL) questionnaire 12

Sexual Anxiety Inventory 12

Sexual optimism scale of Multidimensional Sexual Self Concept Questionnaire (MSSCQ) 12

Sexual Self-Esteem scale 13

Sexual Self-Schema Scale-Revised 13

"20 questions on the impact of genital herpes on non-clinical aspects of the subjects' lives" 13

"25 Herpes-specific questions" 13

Table S1: Critical appraisal checklist for health-related quality-of-life studies 14

Table S2: Quality of the study assessed using the criteria from the methods section of the Consolidated Health Economic Evaluation Reporting Standards (CHEERS) checklist[33].......23

References...24

Search Methodology.

Six databases were searched on January 7 2021. The search looked for mentions of syphilis, quality of life, measures of health state utility, economic evaluations, and economic models. The search limits were from 2000-current, humans, and English language. The search strategy was refined with the project team until the results retrieved reflected the scope of the project. The final Medline search was amended to run across the other databases.

The databases searched were:

1. OvidSP MEDLINE and Epub Ahead of Print, In-Process & Other Non-Indexed Citations, Daily and Versions, 1946 to February 15, 2021
2. OvidSP Embase Classic + Embase, 1947 to January 7, 2020
3. OvidSP EBM Reviews – NHS Economic Evaluation Database, 1st Quarter 2016
4. OvidSP EBM Reviews – Health Technology Assessment, 4th Quarter 2016
5. OvidSP EBM Reviews – Database of Abstracts of Reviews of Effects, 1st Quarter 2016
6. Web of Science Core Collection
    a. Science citation index expanded (1900- present)
    b. Social sciences citation index (1900 to present)
    c. Arts & humanities citation index (1975 to present)
    d. Conference proceedings citation index- Science (1990 to present)
    e. Conference proceedings citation index - Social science & humanities (1990 to present)
    f. Book citation index - science (2005 to present)
    g. Book citation index - Social sciences & humanities (2005 to present)
    h. Current chemical reactions (1985 to present)
    i. Index Chemicus (1993 to present).

Search Results

OvidSP Medline

Database name	Medline
Database platform	OvidSP
Dates of database coverage	1946 to Feb 15, 2021
Date searched	17/02/2021
Searched by	AD
Number of hits	1496

1. herpes.mp. or Herpes Simplex/ or HSV.mp. (75025)
2. exp "Quality of Life"/ or "Quality of Life".mp. or life quality.mp. or hql.mp. or qol.mp. or hrql.mp. or hrqol.mp. (371176)
3. (QALY$ or "Quality adjusted Life Year$").mp. or exp Quality-Adjusted Life Years/ (20548)
4. exp Health Status/ or (health status or health level).mp. (405430)
5. exp Health Status Indicators/ or Health Status Indicators.mp. (315469)
6. (utilit* or close utility analys* or health utilit* or disutility).mp. (222983)
7. exp Health Impact Assessment/ or Health Impact Assessment.mp. (1401)
8. (sf 36 or sf36 or "short form 36" or shortform 36 or sf thirtysix or "sf thirty six" or "short form thirty six" or "short form thirtysix" or "shortform thirty six" or "shortform thirtysix" or (sf 12 or sf12 or "short form 12" or shortform 12 or sf twelve or "short form twelve" or "shortform twelve") or (sf 8 or sf8 or "short form 8" or shortform 8 or sf eight or "short form eight" or "shortform eight") or (sf 6d or sf6d or "short form 6d" or shortform 6d or sf six or "short form six" or "shortform six")).tw. (33732)
9. (euroqol or euro qol or eq5d or eq 5d).mp. (12288)
10. (hui$ or health utilities index).mp. (7888)
11. (standard gamble or time trade off or tto or discrete choice experiment$ or discrete choice model$ or qualitative choice model$).mp. (4325)
12. exp Patient Preference/ or Patient Preference.mp. or (exp Patient Satisfaction/ or Patient Satisfaction.mp.) or preference$.mp. or valuation$.mp. (280045)
13. patient related outcome.mp. (317)
14. cost utility analys*.mp. (3329)
15. exp Cost-Benefit Analysis/ or Cost-Benefit Analys*.mp. or cost effective*.mp. (187532)
16. exp "Costs and Cost Analysis"/ (242495)
17. exp Models, Economic/ or (economic adj3 model$).mp. (18959)
18. markov$.mp. (30587)
19. economic evaluation$.mp. (13325)
20. or/2-19 (1579292)
21. 1 and 20 (2403)
22. limit 21 to (english language and humans and yr="2000 -Current") (1496)

OvidSP Embase Classic + Embase

Database name	Embase Classic + Embase
Database platform	OvidSP
Dates of database coverage	1946 to February 15, 2021
Date searched	17/02/2021
Searched by	AD
Number of hits	4261

1. herpes.mp. or Herpes Simplex/ or HSV.mp. (143118)
2. exp "Quality of Life"/ or "Quality of Life".mp. or life quality.mp. or hql.mp. or qol.mp. or hrql.mp. or hrqol.mp. (645109)
3. (QALY$ or "Quality adjusted Life Year$").mp. or exp Quality-Adjusted Life Years/ (34860)
4. exp Health Status/ or (health status or health level).mp. (294535)
5. exp Health Status Indicators/ or Health Status Indicators.mp. (33634)
6. (utilit* or close utility analys* or health utilit* or disutility).mp. (316205)
7. exp Health Impact Assessment/ or Health Impact Assessment.mp. (5908)
8. (sf 36 or sf36 or "short form 36" or shortform 36 or sf thirtysix or "sf thirty six" or "short form thirty six" or "short form thirtysix" or "shortform thirty six" or "shortform thirtysix" or (sf 12 or sf12 or "short form 12" or shortform 12 or sf twelve or "short form twelve" or "shortform twelve") or (sf 8 or sf8 or "short form 8" or shortform 8 or sf eight or "short form eight" or "shortform eight") or (sf 6d or sf6d or "short form 6d" or shortform 6d or sf six or "short form six" or "shortform six")).tw. (54698)
9. (euroqol or euro qol or eq5d or eq 5d).mp. (22944)
10. (hui$ or health utilities index).mp. (13496)
11. (standard gamble or time trade off or tto or discrete choice experiment$ or discrete choice model$ or qualitative choice model$).mp. (6324)
12. exp Patient Preference/ or Patient Preference.mp. or (exp Patient Satisfaction/ or Patient Satisfaction.mp.) or preference$.mp. or valuation$.mp. (332499)
13. patient related outcome.mp. (485)
14. cost utility analys*.mp. (11440)
15. exp Cost-Benefit Analysis/ or Cost-Benefit Analys*.mp. or cost effective*.mp. (332499)

16. exp "Costs and Cost Analysis"/ (367253)
17. exp Models, Economic/ or (economic adj3 model$).mp. (9227)
18. markov$.mp. (37611)
19. economic evaluation$.mp. (26772)
20. or/2-19 (2115556)
21. 1 and 20 (5562)
22. limit 21 to (english language and humans and yr="2000 -Current") (4261)

OvidSP NHS Economic Evaluation Database

Database name	NHS Economic Evaluation Database
Database platform	OvidSP
Dates of database coverage	1st Quarter 2016
Date searched	17/02/2021
Searched by	AD
Number of hits	55

1. herpes.mp. or Herpes Simplex/ or HSV.mp. (67)
2. exp "Quality of Life"/ or "Quality of Life".mp. or life quality.mp. or hql.mp. or qol.mp. or hrql.mp. or hrqol.mp. (6244)
3. (QALY$ or "Quality adjusted Life Year$").mp. or exp Quality-Adjusted Life Years/ (4752)
4. exp Health Status/ or (health status or health level).mp. (540)
5. exp Health Status Indicators/ or Health Status Indicators.mp. (512)
6. (utilit* or close utility analys* or health utilit* or disutility).mp. (4588)
7. exp Health Impact Assessment/ or Health Impact Assessment.mp. (1)
8. (sf 36 or sf36 or "short form 36" or shortform 36 or sf thirtysix or "sf thirty six" or "short form thirty six" or "short form thirtysix" or "shortform thirty six" or "shortform thirtysix" or (sf 12 or sf12 or "short form 12" or shortform 12 or sf twelve or "short form twelve" or "shortform twelve") or (sf 8 or sf8 or "short form 8" or shortform 8 or sf eight or "short form eight" or "shortform eight") or (sf 6d or sf6d or "short form 6d" or shortform 6d or sf six or "short form six" or "shortform six")).tw. (300)
9. (euroqol or euro qol or eq5d or eq 5d).mp. (731)
10. (hui$ or health utilities index).mp. (114)

11. (standard gamble or time trade off or tto or discrete choice experiment$ or discrete choice model$ or qualitative choice model$).mp. (498)
12. exp Patient Preference/ or Patient Preference.mp. or (exp Patient Satisfaction/ or Patient Satisfaction.mp.) or preference$.mp. or valuation$.mp. (3861)
13. patient related outcome.mp. (0)
14. cost utility analys*.mp. (3136)
15. exp Cost-Benefit Analysis/ or Cost-Benefit Analys*.mp. or cost effective*.mp. (14957)
16. exp "Costs and Cost Analysis"/ (14740)
17. exp Models, Economic/ or (economic adj3 model$).mp. (1833)
18. markov$.mp. (2735)
19. economic evaluation$.mp. (17190)
20. or/2-19 (17397)
21. 1 and 20 (67)
22. limit 21 to (english language and humans and yr="2000 -Current") (55)

OvidSP Health Technology Assessment

Database name	Health Technology Assessment
Database platform	OvidSP
Dates of database coverage	4th Quarter 2016
Date searched	17/02/2021
Searched by	AD
Number of hits	0

1. herpes.mp. or Herpes Simplex/ or HSV.mp. (15)
2. exp "Quality of Life"/ or "Quality of Life".mp. or life quality.mp. or hql.mp. or qol.mp. or hrql.mp. or hrqol.mp. (787)
3. (QALY$ or "Quality adjusted Life Year$").mp. or exp Quality-Adjusted Life Years/ (214)
4. exp Health Status/ or (health status or health level).mp. (71)
5. exp Health Status Indicators/ or Health Status Indicators.mp. (29)
6. (utilit* or close utility analys* or health utilit* or disutility).mp. (239)
7. exp Health Impact Assessment/ or Health Impact Assessment.mp. (7)

8. (sf 36 or sf36 or "short form 36" or shortform 36 or sf thirtysix or "sf thirty six" or "short form thirty six" or "short form thirtysix" or "shortform thirty six" or "shortform thirtysix" or (sf 12 or sf12 or "short form 12" or shortform 12 or sf twelve or "short form twelve" or "shortform twelve") or (sf 8 or sf8 or "short form 8" or shortform 8 or sf eight or "short form eight" or "shortform eight") or (sf 6d or sf6d or "short form 6d" or shortform 6d or sf six or "short form six" or "shortform six")).tw. (13)
9. (euroqol or euro qol or eq5d or eq 5d).mp. (15)
10. (hui$ or health utilities index).mp. (1)
11. (standard gamble or time trade off or tto or discrete choice experiment$ or discrete choice model$ or qualitative choice model$).mp. (7)
12. exp Patient Preference/ or Patient Preference.mp. or (exp Patient Satisfaction/ or Patient Satisfaction.mp.) or preference$.mp. or valuation$.mp. (257)
13. patient related outcome.mp. (0)
14. cost utility analys*.mp. (23)
15. exp Cost-Benefit Analysis/ or Cost-Benefit Analys*.mp. or cost effective*.mp. (1946)
16. exp "Costs and Cost Analysis"/ (1198)
17. exp Models, Economic/ or (economic adj3 model$).mp. (136)
18. markov$.mp. (11)
19. economic evaluation$.mp. (499)
20. or/2-19 (3343)
21. 1 and 20 (2)
22. limit 21 to (english language and humans and yr="2000 -Current") (0)

OvidSP Database of Abstracts of Reviews of Effects

Database name	Database of Abstracts of Reviews of Effects
Database platform	OvidSP
Dates of database coverage	1st Quarter 2016
Date searched	17/02/2021
Searched by	AD
Number of hits	12

1. herpes.mp. (54)

2. HSV.mp. (6)
3. ("Quality of Life" or life quality or hql or qol or hrql or hrqol).mp. (2214)
4. (QALY$ or "Quality adjusted Life Year$").mp. (156)
5. (health status or health level).mp. (380)
6. Health Status Indicators.mp. (44)
7. (utilit* or close utility analys* or health utilit* or disutility).mp. (330)
8. Health Impact Assessment.mp. (1)
9. (sf 36 or sf36 or "short form 36" or shortform 36 or sf thirtysix or "sf thirty six" or "short form thirty six" or "short form thirtysix" or "shortform thirty six" or "shortform thirtysix" or (sf 12 or sf12 or "short form 12" or shortform 12 or sf twelve or "short form twelve" or "shortform twelve") or (sf 8 or sf8 or "short form 8" or shortform 8 or sf eight or "short form eight" or "shortform eight") or (sf 6d or sf6d or "short form 6d" or shortform 6d or sf six or "short form six" or "shortform six")).tw. (125)
10. (euroqol or euro qol or eq5d or eq 5d).mp. (22)
11. (hui$ or health utilities index).mp. (1)
12. (standard gamble or time trade off or tto or discrete choice experiment$ or discrete choice model$ or qualitative choice model$).mp. (2)
13. (Patient Preference or Patient Satisfaction or preference$ or valuation$).mp. (964)
14. patient related outcome.mp. (4)
15. cost utility analys*.mp. (33)
16. (Cost-Benefit Analys* or cost effective*).mp. (1626)
17. (economic adj3 model*).mp. (93)
18. markov$.mp. (73)
19. economic evaluation$.mp. (335)
20. 1 or 2 (54
21. or/3-19 (4633)
22. 20 and 21 (12)

Web of Science

Database name	Web of Science Core Collection
Database platform	Web of Science (last updated: 2021-02-16)
Dates of database coverage	Science citation index expanded (1900-present)

	Social sciences citation index (1900 to present)<br>Arts & humanities citation index (1975 to present)<br>Conference proceedings citation index-Science (1990 to present)<br>Conference proceedings citation index - Social science & humanities (1990 to present)<br>Book citation index - science (2005 to present)<br>Book citation index - Social sciences & humanities (2005 to present)<br>Emerging Sources Citation Index (ESCI) --2015-present<br>Current chemical reactions (1985 to present)<br>Index Chemicus (1993 to present)
Date searched	17/02/2021
Searched by	AD
Number of hits	2667

1. ALL=(herpes or HSV) (77655)
2. TS=(quality of life or quality adjusted life year* or QALY*) (588073)
3. TS=(health status or health state* or health status indicator*) (557130)
4. TS=(utility* or health utility* or disutility) (389592)
5. TS=(health impact assessment*) (58470)
6. TS=(sf 36 or sf36 or "short form 36" or shortform 36 or sf thirtysix or "sf thirty six" or "short form thirty six" or "short form thirtysix" or "shortform thirty six" or "shortform thirtysix" or (sf 12 or sf12 or "short form 12" or shortform 12 or sf twelve or "short form twelve" or "shortform twelve") or (sf 8 or sf8 or "short form 8" or shortform 8 or sf eight or "short form eight" or "shortform eight") or (sf 6d or sf6d or "short form 6d" or shortform 6d or sf six or "short form six" or "shortform six")) (47398)
7. TS=(euroqol or euro qol or eq5d or eq 5d or hui$ or health utilities index or standard gamble or time trade off or tto or discrete choice experiment$ or discrete choice model$ or qualitative choice model$) (68842)
8. TS=(patient preference$ or patient satisfaction or preference$ or valuation$ or patient related outcome$) (739076)

9. TS=(cost utility analys?s or cost benefit analys?s or cost analys?s or economic evaluation$) (511623)
10. TS=(economic model* or economic NEAR/3 model* or markov$) (392269)
11. #2 OR #3 OR #4 OR #5 OR #6 OR #7 OR #8 OR #9 OR #10 (2869936)
12. (#1 AND #11) AND language: (English) AND Timespan=2000-2021 (2667)

Survey instruments for evaluating genital herpes quality of life

This section provides a summary of the survey instruments used to measure genital herpes related quality of life.

Female Sexual Function Index (FSFI)

FSFI is a self-administered questionnaire to assess sexual well-being developed in 2000 by Raymond Rosen, and a French version has been validated.[1,2] A 6-item questionnaire allows the assessment of female sexual function (desire, arousal, lubrication, orgasm, satisfaction and pain) over the past four weeks. The total score ranges between 2 and 36; higher scores are associated with a lower degree of female sexual dysfunction (FSD). The choice of a pertinent threshold to define FSD is discussed in the literature, but a consensus seems to be found for values of less than 23.[3-5]

Global measure of sexual satisfaction-revised (GMSEX-R)

The GMSEXR assesses sexual satisfaction with sexual relationship(s) for individuals with or without a current partner.[6] Responses are made on five 7-point scales: good–bad, pleasant–unpleasant, positive– negative, satisfying–unsatisfying, and valuable–worthless. Responses were summed with higher scores indicating greater sexual satisfaction. GMSEX-R showed convergent validity, supported by significant correlations with other measures of sexual satisfaction as well as measures of sexual rewards and costs among individuals in married/cohabiting and dating relationships.[6] The internal consistency of the GMSEX-R is high.[7,8]

Herpes Outbreak Impact Questionnaire (HOIQ)

HOIQ is a 14-item assessment of daily function (disability) specific to genital herpes outbreaks. The HOIQ was designed to complement the Herpes Symptom Checklist (HSC) but can be used alone.[9] The instrument was designed to be responsive to change in impact over a short period of time and intended to be used during outbreaks only.[9] It uses a 4-point response scale (not at all, a little, a lot, very much).[9] It was shown to be acceptable, relevant, and understandable to patients in the UK and the US.[9]

Herpes Symptom Checklist (HSC)

The HSC is a 13-item questionnaire with a 4-point severity response scale (none, mild, moderate, and severe) to rate the presence and severity of symptoms on the day of completion.[9] The instrument was designed to be responsive to change in impact over a short period of time and intended to be used during outbreaks only.[9] The HSC was designed to be complementary to the HOIQ but can also be used alone.[9] It was shown to be acceptable, relevant, and understandable to patients in the UK and the US.[9]

Recurrent Genital Herpes Quality of Life (RGHQoL) questionnaire

The RGHQoL consists of 20 statements that take several minutes to complete.[10] Patients respond to each statement by indicating the level of their own limitation on a 4-point Likert scale with scores ranging from 0 (maximum limitation) to 3 (minimum limitation). The RGHQoL is founded on the principle that quality of life relates to the ability and capacity of an individual to satisfy his or her human needs.[11] It assesses the overall impact of infection on the individual's QoL in the long term.[9] Versions have been created for the UK, USA, Italy, Germany, France and Denmark.[11] The RGHQoL was shown to have good reliability.[11]

Sexual Anxiety Inventory

The Sexual Anxiety Inventory is a 28-item measure that assesses the extent to which an individual feels or would feel anxious when engaged in different sexual activities (sexual anxiety).[7] It considers negative affect associated with sexual activity.[7] Responses are on a 7-point Likert scale ranging from relaxing, calming (-1) to always causes anxiety, extremely anxiety producing (5). Total scores are from -25 to 125, with higher scores indicating more anxiety. The scale is reported to have construct validity and high internal consistency.[7]

Sexual optimism scale of Multidimensional Sexual Self Concept Questionnaire (MSSCQ)

The MSSCQ is an objective self-report instrument used to measure 20 psychological aspects of human sexuality.[12] These include sexual anxiety, sexual self-efficacy, sexual-consciousness, motivation to avoid risky sex, chance/luck sexual control, sexual-assertiveness, sexual-optimism, sexual problem self-blame, sexual-monitoring, sexual-motivation, sexual problem management, sexual-esteem, sexual-satisfaction, power-other sexual control, sexual self-schemata, fear-of-sex, sexual problem prevention, sexual-

depression, internal-sexual-control.[12] There was evidence for both the internal consistency and test-retest stability of the MSSCQ subscales, as well as preliminary evidence for its anticipated factor structure and initial evidence for the concurrent validity.[12] Sexual optimism is defined as the expectation that the future sexual aspects of one's life will be positive and rewarding, which MSSCQ can measure. The values of individual responses (range of 0–4) were averaged, and a higher score suggested greater optimism.[13] A Farsi version of MSSCQ is also available and was applied in youth living in Isfahan.[14]

Sexual Self-Esteem scale

The Sexual Self-Esteem Scale (10-item measure, Responses range from strongly disagree (1) to strongly agree (4), with higher scores indicating higher sexual self-esteem).[7] No other details of its psychometric properties were found.

Sexual Self-Schema Scale-Revised

Sexual Self-Schema Scale-Revised assesses the cognitive representation and sexual self-esteem the affective representation of the sexual self.[7] It has 35 adjectives on a 7-point scale with higher scores indicate a more positive self-schema. Participants rate the extent to which each adjective (e.g., romantic) is characteristic of their sexual self on a 7-point scale ranging from not at all descriptive of me (1) to very descriptive of me (8). Good internal consistency and validity have been reported.[15]

"20 questions on the impact of genital herpes on non-clinical aspects of the subjects' lives"

We could neither find further details about this tool nor its psychometric properties. This may be a section of a study-specific questionnaire.[16]

"25 Herpes-specific questions"

"25 herpes specific questions" is a measure to assess the effect of genital herpes on HRQOL, scoring on a 4-point scale (ranges from 0-100). Items for the measure were initially developed in the UK by Hunt and McKenna using the Needs-Based approach. It is unclear about dimensions and items are included. One study in the USA testing the psychometric properties shows that the measure is valid and reliable and can be used with confidence.[17]

Table S1: Critical appraisal checklist for health-related quality-of-life studies

Criteria adapted from references	Issues to consider	Bartlett[18]	Bodsworth[19]	Fife[20]	Fisman[21]	Foster[7]	Langley[22]	Mehta[23]	Meyer[13]	Patel[10]	Raj[24]
Conceptual											
Study objectives	Were the objectives of the study clearly stated? HRQoL primary or secondary outcome?	Yes, secondary	Yes, secondary	Yes, secondary	Yes, primary	Yes, primary	Yes, primary	Yes, primary	Yes, primary	Yes, primary	Yes, primary
HRQoL instrument	Was a reason provided to justify the instrument selected? Was a validated tool used to assess QoL?	Yes, yes	Yes, yes	No, yes	Yes, yes	Yes, yes	No, yes	Yes, no	Yes, yes	Yes, yes	Yes, yes
Methodology											
Study design	Was the design clearly described? (e.g. cohort, cross-sectional, survey)	Yes	Yes	Yes	Yes	Yes	Yes	Yes	Yes	Yes	No
Respondent selection and recruitment	Was the sampling method for recruitment of participants adequately described?	Yes	Yes	Yes	Yes	Yes	No	Yes	No	Yes	No

Inclusion/ exclusion criteria	Are inclusion/ exclusion criteria clearly described? Do these exclude any individuals that might be relevant (e.g. very elderly aged >80 years old)?	Yes	Yes	No	No	Yes	Yes	Yes	Yes	Yes	Yes
Participant characteristics	Were characteristics of participants clearly described (demographics and clinical variables)?	Yes	Yes	Yes	Yes	Yes	Unclear	Yes	Yes	No	Yes
Sample size	Was the sample size used appropriately justified?	Yes	Yes	No	No	No	Unclear	Yes	Yes	No	No
Instrument administration	Is it reported who and/or in which clinical setting the instrument was administered?	Yes	Yes	No	No	Yes	Yes	Yes	Yes	Yes	No
Timing of assessments	Is the timing of assessments reported? (e.g. baseline and/or at follow-up or after treatment	Yes	Yes	Yes	No	Yes	Yes	Yes	Yes	Yes	No
Results											

Response rates to instrument used	Are response rates reported and if so, are the rates likely to be a threat to validity?	Yes	No	No	Yes	Yes	No	No	No	No	No
Loss to follow-up	Is the loss to follow-up reported and are reasons given? Are these likely to threaten the validity of results (e.g. characteristicss of non-responders different to responders)?	Yes, no	Yes, unclear	Yes, unclear	Yes, unclear	Yes, unclear	No	Yes, unclear	No	No	No
Missing data	Are the levels of missing data reported? How are they dealt with? Could this threaten the validity of results?	No	No	No	No	Yes, no	No	No	Yes, no	No	No
Statistical analysis	Were appropriate statistical methods used?	Yes	Yes	Yes	Yes	Yes	Unclear	Yes	Yes	Yes	Yes
Interpretation											
Study findings	Were the key findings of the study clearly stated?	Yes	Yes	Yes	Yes	Yes	Yes	Yes	Yes	Yes	Yes
Study limitations	Were limitations of the study clearly described?	Yes	Yes	No	Yes	Yes	Unclear	Yes	No	Yes	No

Table S1: Critical appraisal checklist for health-related quality-of-life studies (continued)

Criteria adapted from references	Issues to consider	Richards[25]	Romanowski[26]	Spencer[27]	Taboulet[28]	Wild[17]	Wylomansk[29]	Salomon[30]	Salomon[31]	James[32]
	Conceptual									
Study objectives	Were the objectives of the study clearly stated? HRQoL primary or secondary outcome?	Yes, secondary	Yes, secondary	Yes, primary	Yes, primary	Yes, secondary	Yes, primary	Yes, primary	Yes, primary	Yes, secondary
HRQoL instrument	Was a reason provided to justify the instrument selected? Was a validated tool used to assess QoL?	Yes, unclear	No, yes	Yes, yes	Yes, yes	Yes, yes	Yes, yes	No, no	No, no	No, no

	Methodology									
Study design	Was the design clearly described? (e.g. cohort, cross-sectional, survey)	Yes	Yes	No	Yes	Yes	Yes	No	No	No
Respondent selection and recruitment	Was the sampling method for recruitment of participants adequately described?	Yes	Yes	Yes	Yes	Yes	Yes	Yes	Yes	No
Inclusion/exclusion criteria	Are inclusion/exclusion criteria clearly described? Do these exclude any individuals	Yes	Yes	No	Yes	Yes	Yes	No	No	No

	that might be relevant (e.g. very elderly aged >80 years old)?									
Participant characteristics	Were characteristics of participants clearly described (demographics and clinical variables)?	Yes	Yes	Yes	Yes	Yes	Yes	Yes	Yes	No
Sample size	Was the sample size used appropriately justified?	Yes	Yes	No	No	No	No	No	No	No
Instrument administration	Is it reported who and/or in which clinical setting the	Yes	Yes	Yes	Yes	No	Yes	Yes	Yes	Yes

	instrument was administered?									
Timing of assessments	Is the timing of assessments reported? (e.g. baseline and/or at follow-up or after treatment	Yes	Yes	No	Yes	Yes	Yes	N/A	N/A	N/A
	Results									
Response rates to instrument used	Are response rates reported and if so, are the rates likely to be a threat to validity?	Yes, no	Yes, no	Yes, no	Yes, yes	Yes, no	No	Yes, unclear	Yes, unclear	No
Loss to follow-up	Is the loss to follow-up reported and are reasons given? Are	Yes, no	Yes, no	No	Yes, unclear	Yes	No	N/A	N/A	N/A

	these likely to threaten the validity of results (e.g. characteristics of non-responders different to responders)?									
Missing data	Are the levels of missing data reported? How are they dealt with? Could this threaten the validity of results?	Yes. Person-level mean substitution were used to estimate missing. Unclear if it would	No, not stated, not clear.	Yes, 2.5% so low and distributed, not dealt with, not likely to threaten validity	No, not stated, not clear.	Yes, not stated how to deal with missing, unclear if the missing would threaten the validity	No, not stated, not clear.	Yes, unclear, possibly	No	No

		threaten the validity								
Statistical analysis	Were appropriate statistical methods used?	Yes	Yes	Yes	Yes	yes	Yes	Unclear	Unclear	Unclear
	Interpretation									
Study findings	Were the key findings of the study clearly stated?	Yes	Yes	Yes	Yes	Yes	Yes	Yes	Yes	Yes
Study limitations	Were limitations of the study clearly described?	No	No	No	Yes	No	Yes	Yes	Yes	Yes

Table S2: Quality of the study assessed using the criteria from the methods section of the Consolidated Health Economic Evaluation Reporting Standards (CHEERS) checklist[33]

Study	Methods									
	**Target population and subgroups**	**Setting and location**	**Study perspective**	**Time horizon**	**Discount rate**	**Estimating resources and costs**	**Currency, price date, and conversion**	**Choice of model**	**Assumptions**	**Analytical methods**	**Final Score /10**
Baker[34]	1	1	1	0	1	1	1	0	1	1	8
Caviness[35]	1	1	1	0	1	1	1	0	1	1	8
Chatroux[36]	1	1	1	1	1	1	0	0	1	1	8
Little[37]	1	1	1	0	1	1	1	0	1	1	8
Smith[38]	0	0	1	1	1	0	0	0	0	0	3
Thung[39]	1	1	0	0	1	0	1	0	1	0	5
Tuite[40]	1	1	0	0	1	1	1	1	1	1	8

References

1. Rosen CBJHSLCMRSDFRDAR. The Female Sexual Function Index (FSFI): A Multidimensional Self-Report Instrument for the Assessment of Female Sexual Function. *Journal of Sex & Marital Therapy* 2000; **26**(2): 191-208.
2. Wylomanski S, Bouquin R, Philippe H-J, et al. Psychometric properties of the French Female Sexual Function Index (FSFI). *Quality of Life Research* 2014; **23**(7): 2079-87.
3. Aslan E, Beji NK, Gungor I, Kadioglu A, Dikencik BK. Prevalence and risk factors for low sexual function in women: a study of 1,009 women in an outpatient clinic of a university hospital in Istanbul. *The Journal of Sexual Medicine* 2008; **5**(9): 2044-52.
4. Chedraui P, Pérez-López FR, Mezones-Holguin E, San Miguel G, Avila C, Collaborative Group for Research of the Climacteric in Latin A. Assessing predictors of sexual function in mid-aged sexually active women. *Maturitas* 2011; **68**(4): 387-90.
5. Esposito K, Ciotola M, Giugliano F, et al. Association of body weight with sexual function in women. *International journal of impotence research* 2007; **19**(4): 353-7.
6. Lawrance K-a, Byers ES, Cohen JN. Interpersonal exchange model of sexual satisfaction questionnaire. *Sexuality-related measures: A compendium* 1998; **2**: 525-30.
7. Foster LR, Byers ES. Predictors of the Sexual Well-being of Individuals Diagnosed with Herpes and Human Papillomavirus. *Archives of Sexual Behavior* 2016; **45**(2): 403-14.
8. Renaud CA, Byers ES. Positive and negative sexual cognitions: Subjective experience and relationships to sexual adjustment. *The Journal of Sex Research* 2001; **38**(3): 252-62.
9. Doward LC, McKenna SP, Meads DM, Kahler K, Frech F. The Development of the Herpes Symptom Checklist and the Herpes Outbreak Impact Questionnaire. *Value in Health* 2009; **12**(1): 139-45.
10. Patel R, Tyring S, Strand A, Price M, Grant DJSti. Impact of suppressive antiviral therapy on the health related quality of life of patients with recurrent genital herpes infection. 1999; **75**(6): 398-402.
11. Doward LC, McKenna SP, Kohlmann T, et al. The international development of the RGHQoL: a quality of life measure for recurrent genital herpes. *Qual Life Res* 1998; **7**(2): 143-53.
12. Snell WE, Fisher TD, Walters AS. The Multidimensional Sexuality Questionnaire: An objective self-report measure of psychological tendencies associated with human sexuality. *Annals of Sex Research* 1993; **6**(1): 27-55.
13. Meyer JL, Crosby RA, Whittington WL, et al. The psychosocial impact of serological herpes simplex type 2 testing in an urban HIV clinic. *Sexually Transmitted Infections* 2005; **81**(4): 309-15.
14. Ziaei T, Khoei EM, Salehi M, Farajzadegan Z. Psychometric properties of the Farsi version of modified Multidimensional Sexual Self-concept Questionnaire. *Iran J Nurs Midwifery Res* 2013; **18**(6): 439-45.
15. Andersen BL, Cyranowski JM, Espindle D. Men's sexual self-schema. *J Pers Soc Psychol* 1999; **76**(4): 645-61.
16. Taboulet F, Halioua B, Malkin JE. Quality of life and use of health care among people with genital herpes in France. *Acta dermato-venereologica* 1999; **79**(5).
17. Wild D, Patrick D, Johnson E, Berzon R, Wald A. Measuring health-related quality of life in persons with genital herpes. *Quality of life research : an international journal of quality of life aspects of treatment, care and rehabilitation* 1995; **4**(6): 532-9.
18. Bartlett BL, Tyring SK, Fife K, et al. Famciclovir treatment options for patients with frequent outbreaks of recurrent genital herpes: The RELIEF trial. *Journal of Clinical Virology* 2008; **43**(2): 190-5.

19. Bodsworth N, Bloch M, McNulty A, et al. 2-day versus 5-day famciclovir as treatment of recurrences of genital herpes: results of the FaST study. *Sexual Health* 2008; **5**(3): 219-25.
20. Fife KH, Almekinder J, Ofner S. A comparison of one year of episodic or suppressive treatment of recurrent genital herpes with valacyclovir. *Sex Transm Dis* 2007; **34**(5): 297-301.
21. Fisman DN. Health related quality of life in genital herpes: a pilot comparison of measures. *Sex Transm Infect* 2005; **81**(3): 267-70.
22. Langley PC, Freedman D, Wagner JS, Gupta S. The experience of external genital warts and genital herpes on quality of life. *Value in Health* 2010; **13 (7)**: A403.
23. Mehta SD, Nordgren RK, Agingu W, et al. Sexual Quality of Life and Association With HIV and Sexually Transmitted Infections Among a Cohort of Heterosexual Couples in Kenya. *J Sex Med* 2018; **15**(10): 1446-55.
24. Raj R, Sreenivas V, Mehta M, Gupta S. Health-related quality of life in Indian patients with three viral sexually transmitted infections: herpes simplex virus-2, genital human papilloma virus and HIV. *Sex Transm Infect* 2011; **87**(3): 216-20.
25. Richards J, Scholes D, Caka S, et al. HSV-2 serologic testing in an HMO population: Uptake and psychosocial sequelae. *Sexually Transmitted Diseases* 2007; **34**(9): 718-25.
26. Romanowski B, Marina RB, Roberts JN. Patients' preference of valacyclovir once-daily suppressive therapy versus twice-daily episodic therapy for recurrent genital herpes: A randomized study. *Sexually Transmitted Diseases* 2003; **30**(3): 226-31.
27. Spencer B, Leplège A, Ecosse E. Recurrent genital herpes and quality of life in France. *Qual Life Res* 1999; **8**(4): 365-71.
28. Taboulet F, Halioua B, Malkin JE. Quality of life and use of health care among people with genital herpes in France. *Acta Derm Venereol* 1999; **79**(5): 380-4.
29. Wylomanski S, Bouquin R, Hanf M, et al. Sexual well-being in patients with vulvar disease: Results from a preliminary prospective matched case-control study. *European Journal of Obstetrics and Gynecology and Reproductive Biology* 2015; **194**: 106-10.
30. Salomon JA, Vos T, Hogan DR, et al. Common values in assessing health outcomes from disease and injury: disability weights measurement study for the Global Burden of Disease Study 2010. *The Lancet* 2012; **380**(9859): 2129-43.
31. Salomon JA, Haagsma JA, Davis A, et al. Disability weights for the Global Burden of Disease 2013 study. *The Lancet Global Health* 2015; **3**(11): e712-e23.
32. GBD 2017 Disease and Injury Incidence and Prevalence Collaborators. Global, regional, and national incidence, prevalence, and years lived with disability for 354 diseases and injuries for 195 countries and territories, 1990-2017: a systematic analysis for the Global Burden of Disease Study 2017. *Lancet* 2018; **392**(10159): 1789-858.
33. CHEERS Checklist - Items to include when reporting economic evaluations of health interventions 2013. https://www.equator-network.org/wp-content/uploads/2013/04/Revised-CHEERS-Checklist-Oct13.pdf2020).
34. Baker D, Brown Z, Hollier LM, et al. Cost-effectiveness of herpes simplex virus type 2 serotogic testing and antiviral therapy in pregnancy. *American Journal of Obstetrics and Gynecology* 2004; **191**(6): 2074-84.
35. Caviness AC, Demmler GJ, Swint JM, Cantor SB. Cost-effectiveness analysis of herpes simplex virus testing and treatment strategies in febrile neonates. *Archives of pediatrics & adolescent medicine* 2008; **162**(7): 665-74.
36. Chatroux IC, Hersh AR, Caughey AB. Herpes Simplex Virus Serotyping in Pregnant Women With a History of Genital Herpes and an Outbreak in the Third Trimester of Pregnancy: A Cost-Effectiveness Analysis. *Obstetrics and gynecology* 2021; **137**(1): 63-71.

37. Little SE, Caughey AB. Acyclovir prophylaxis for pregnant women with a known history of herpes simplex virus: a cost-effectiveness analysis. *Am J Obstet Gynecol* 2005; **193**(3 Pt 2): 1274-9.
38. Smith KJ, Cook RL, Downs JS, Roberts MS. Cost-effectiveness of management strategies for chronic recurrent genital herpes: A pilot analysis. *Journal of General Internal Medicine* 2000; **15**: 4-.
39. Thung SF, Grobman WA. The cost-effectiveness of routine antenatal screening for maternal herpes simplex virus-1 and -2 antibodies. *Am J Obstet Gynecol* 2005; **192**(2): 483-8.
40. Tuite AR, McCabe CJ, Ku J, Fisman DN. Projected cost-savings with herpes simplex virus screening in pregnancy: towards a new screening paradigm. *Sexually Transmitted Infections* 2011; **87**(2): 141.
